# Surgical and Peri-Operative Considerations for Brain Metastases

**DOI:** 10.3389/fonc.2021.662943

**Published:** 2021-05-05

**Authors:** Saksham Gupta, Hassan Dawood, Alexandra Giantini Larsen, Luis Fandino, Erik H. Knelson, Timothy R. Smith, Eudocia Q. Lee, Ayal Aizer, Ian F. Dunn, Wenya Linda Bi

**Affiliations:** ^1^ Center for Skull Base and Pituitary Surgery, Department of Neurosurgery, Brigham and Women’s Hospital, Harvard Medical School, Boston, MA, United States; ^2^ Department of Neurosurgery, Weill Cornell Medical College, New York City, NY, United States; ^3^ Department of Orthopedic Surgery, University of Utah Health Science Center, Salt Lake City, UT, United States; ^4^ Department of Medical Oncology, Dana Farber Cancer Institute, Harvard Medical School, Boston, MA, United States; ^5^ Center for Neuro-Oncology, Dana Farber Cancer Institute, Harvard Medical School, Boston, MA, United States; ^6^ Department of Radiation Oncology, Brigham and Women’s Hospital, Harvard Medical School, Boston, MA, United States; ^7^ Department of Neurosurgery, Oklahoma University Health Sciences Center, Oklahoma City, OK, United States

**Keywords:** brain metastasis, surgical risk, readmission, reoperation, complications, mortality

## Abstract

**Introduction:**

Brain metastases are the most common brain tumors in adults, whose management remains nuanced. Improved understanding of risk factors for surgical complications and mortality may guide treatment decisions.

**Methods:**

A nationwide, multicenter analysis was conducted with a retrospective cohort. Adult patients in the 2012-2015 American College of Surgeons National Surgical Quality Improvement Project (ACS NSQIP) databases who received a craniotomy for resection of brain metastasis were included.

**Results:**

3500 cases were analyzed, of which 17% were considered frail and 24% were infratentorial. The most common 30-day medical complications were venous thromboembolism (3%, median time-to-event [TTE] 4.5 days), pneumonia (4%, median TTE 6 days), and urinary tract infections (2%, median TTE 5 days). Reoperation and unplanned readmission occurred in 5% and 12% of patients, respectively. Infratentorial approach and frailty were associated with reoperation before discharge (OR 2.0 for both; p=0.01 and p=0.03 respectively), but not after discharge. Infratentorial approaches conferred heightened risk for readmission for hydrocephalus (OR 5.1, p=0.02) and reoperation for cerebrospinal fluid diversion (OR 7.1, p<0.001).Overall 30-day mortality was 4%, with nearly three-quarters occurring after discharge. Pre-frailty and frailty were associated with increased odds for post-discharge mortality (OR 1.7 and 2.7, p<0.05), but not pre-discharge mortality. We developed a model to identify pre-/peri-operative variables associated with death, including frailty, thrombocytopenia, and high American Society of Anesthesiologists score (AUROC 0.75).

**Conclusions:**

Optimization of metrics contributing to patient frailty and heightened surveillance in patients with infratentorial metastases may be considered in the peri-operative period.

## Introduction

Solid tumor brain metastasis is associated with high morbidity and mortality. The overall annual incidence of brain metastasis amongst cancer patients ranges from 2-10% and varies greatly by primary cancer origin, tumor stage, and demographic factors including race and age (1–3). The most common sites of origin for brain metastasis are lung (39-56%), breast (13-30%), and melanoma (6-11%) (1, 4). The incidence of brain metastasis has increased over the past 20 years in parallel with improved metastasis detection and treatment of the primary cancer; as patients live longer, their risk of brain metastasis increases (4, 5). Among patients with brain metastases, median survival ranges from 3 months for pancreatic cancer, to 6-9 months for melanoma, to 6-13 months or longer for lung and breast cancer (6–9).

Standard management for brain metastases involves, in general, radiation therapy with or without surgical resection. Surgical resection of brain metastases is typically indicated for isolated or oligo-metastatic disease, symptomatic or large tumors causing mass effect, and radiation-resistant tumors (10, 11). Resecting a metastasis may reduce symptoms, prevent neurological damage, and increase survival in select cases (12–15). However, surgery exposes a patient to the risks of an operation, hospitalization, as well as the recovery period. Especially for patients with finite life expectancy from their malignancy, the expected benefits should outweigh the potential risks to optimize quality of life.

We sought to investigate factors that contribute to adverse events following resection of brain metastases in order to inform selection of patients for surgery. We focused on tumor location and frailty in this analysis. Infratentorial tumors can present higher surgical risk due to their proximity to the brainstem and vital neurovascular structures. Frailty quantifies a patient’s chronic disease burden and has been independently associated with poorer health outcomes in nonsurgical patients and adverse postoperative outcomes in other surgical fields (16–20).

## Materials and Methods

### Data Source

The American College of Surgeons National Surgical Quality Improvement Project (NSQIP) 2012-2015 databases were obtained for this analysis. These databases contain validated, multi-institutional data collected in a standardized method across institutions by trained coders (21). The NSQIP databases contain cases from private and academic centers. In 2012, 374 hospitals provided data, which rose to 603 by 2015.

### Inclusion and Exclusion Criteria

The NSQIP database was filtered by relevant criteria. Inclusion required a billing code for brain metastasis (ICD9 198.3 or ICD10 C79.31), and a neurosurgical CPT code of 61510, 61518, 61520, 61521, 61526, or 61530. Patients were included if they were at least 18 years old, had a neurosurgeon as the primary attending surgeon for their case, and received a procedure under general anesthesia. These criteria yielded 3567 cases. Of these, 67 were excluded for operative time under 30 minutes or post-operative length of stay of 0 days since these were assumed to be part of an aborted or miscoded procedure.

### Patient Stratification 

Pertinent preoperative and perioperative co-variates were obtained for each case. Sex, age, body mass index (BMI), case urgency, pre-operative co-morbidities, American Society of Anesthesiologist (ASA) class, and pre-operative laboratory abnormalities were included. Tumor location was extrapolated based on the associated current procedural terminology (CPT) code, with infratentorial (CPT 61518, 61520, 61521, 61526, or 61530) and supratentorial (CPT 61510) strata. These codes were initially derived to stratify procedures for billing purposes. Operative time was binned into quartiles.

The modified frailty index (mFI) with 5 co-variates (mFI-5) was calculated for each case as an estimate of patients’ chronic disease burden with one point assigned for congestive heart failure (CHF), chronic obstructive pulmonary disease (COPD), diabetes mellitus (DM), hypertension (HTN), and non-independent functional status. This scale is a modification of the validated mFI-11, with strong concordance with the mFI-11 in prior nationwide analyses (16, 22). A mFi-5 score of 0 was considered non-frail, 1 as pre-frail, and 2+ as frail.

NSQIP databases encode postoperative outcomes up to 30 days, including surgical site infections (SSIs), wound dehiscence, pneumonia, reintubation, deep vein thrombosis (DVT)/pulmonary embolism (PE), urinary tract infections (UTI), cerebrovascular accidents (CVAs), cardiac arrests, myocardial infarction (MI), transfusions, sepsis, extended length of stay defined as over 1 week, reoperation, unplanned readmission, and death. Outcomes of interest were further stratified by whether they occurred during the index hospitalization or after initial discharge. Analyses of post-discharge complications excluded patients who died during the index hospitalization.

Complications encoded on the day of discharge were considered post-discharge as they would likely have prolonged hospitalizations had they occurred prior to a discharge. In contrast, death that was encoded on the day of discharge was considered pre-discharge given that a critical patient at risk of death would likely not have been discharged that same day.

### Statistical Analysis

The R Package for Statistical Computing version 3.5.1 was utilized for all analyses. Cases with missing co-variate data were binned into “missing” categories and treated as a separate independent variable during all analyses. The impact of frailty and tumor location on all postoperative outcomes was calculated as odds ratios (OR) through multivariate regression with adjustment for sex, age, BMI, ASA class, transfer from outside hospital, tumor location, smoking, steroid usage, case urgency, pre-operative lab abnormalities, operative time, use of operative microscope and stereotactic navigation. A two-sided probability value (p) of under 0.05 was utilized as the threshold for statistical significance.

A predictive model for 30-day death was also developed. A univariate screen of all co-variates associated with death, excluding any co-variates with fewer than 0.1% of any level associated with a death, was conducted using a χ^2^ statistical significance cut-off of p < 0.10. The screened co-variates were included in a multivariate logistic regression with backwards stepwise variable elimination. The Hosmer-Lemeshow test and area under the receiver-operator curve (AUROC) concordance statistic was utilized to assess calibration and discrimination, respectively. The pre-determined value for an acceptable calibration for the model was p ≥ 0.05.

## Results

### Case Characteristics

Within the study population, 3500 patients with brain metastases were included, with 55% being female **(** **Table 1** **)**. Median age of this cohort was 61 years (Interquartile range [IQR] 54-69). Infratentorial lesions represented 24% of cases. Patients were considered non-frail (n=1672, 48%), pre-frail (n=1245, 36%), or frail (n=583, 17%) as quantified by the mFI-5 index. Most patients were admitted to the same hospital where they presented (n=2722, 78%), and 50% of cases were electively scheduled. Median operative length was 139 minutes (IQR 98-193).

**Table 1:** Clinical Characteristics.

Total cases = 3500		
Sex	N	%
Female	1936	55.3
Age	N	%
18-50	636	18.2
50-65	1619	46.3
65-75	949	27.1
75+	296	8.5
BMI	N	%
Normal	2365	67.6
Class I Obese	611	17.5
Class II Obese	382	10.9
Unknown	142	4.1
Past Medical History	N	%
CHF	17	0.5
COPD	432	12.3
DM	413	11.8
HTN Meds	1455	41.6
Smoker	1042	29.8
Steroid Use	860	24.6
Modified Frailty Index	N	%
0 = Non-Frail	1672	47.8
1 = Pre-Frail	1245	35.6
2+ = Frail	583	16.7
ASA Class	N	%
1-2	394	11.3
3	2323	66.4
4-5	764	21.8
Unknown	19	0.5
Location of Tumor	N	%
Supratentorial	2674	76.4
Infratentorial	826	23.6
Location of Arrival	N	%
Direct Admit	2722	77.8
Transfer	775	22.1
Unknown	3	0.1

ASA, American Society of Anesthesiologists; BMI, body mass index; CHF, congestive heart failure; COPD, chronic obstructive pulmonary disease; DM, Diabetes mellitus; HTN, hypertension; N, Number.

### Postoperative Course

**Figure 1:**
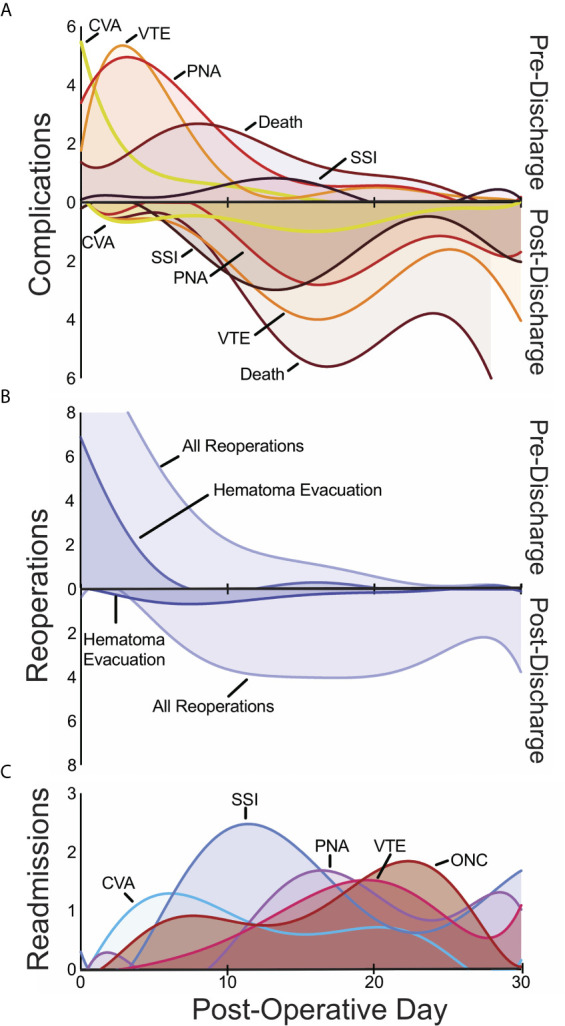
Temporal patterns in 30-day pre- and post-discharge medical complications **(A)**, pre- and post-discharge reoperations **(B)**, and readmissions **(C)** with the day of the index operation defined as post-operative day 0. CSF, cerebrospinal fluid; CVA, cerebrovascular accident; ONC, medical oncologic admission; PNA, pneumonia; SSI, surgical site infection; VTE venous thromboembolism.

We first examined post-operative medical complications and interrogated their temporal patterns in an effort to identify risk factors for poor or unexpected outcomes. Median length of stay following operation was 3 days (IQR 2-5); 14% of patients were hospitalized for longer than 7 days postoperatively. The most frequent postoperative medical complications overall were venous thromboembolism (VTE; including DVTs and PEs), pneumonia, UTI, and sepsis (**Table 2**). Stratifying complications by whether they occurred during index hospitalization or after initial discharge revealed temporal trends for specific complications (**Table 2**). Cardiac events (myocardial infarctions and cardiac arrests) and CVAs tended to occur within 0-2 days postoperatively and prior to discharge (**Figure 1A**). In contrast, the median time to occurrence for in-hospital DVTs, infectious complications, and reoperations tended to be 4-6 days. Complications that arose after discharge occurred with a wide temporal spread (**Figure 1A**). Superficial site infections and sepsis tended to be more common after discharge than before discharge.

**Table 2:** Prevalence of complications stratified by index hospitalization.

	Total	Pre-Discharge			Post-Discharge		
	%	%	Median (days)	IQR	%	Median (days)	IQR
Superficial Site Infection	1.49	0.26	13	10-14	1.23	14	12-21.52
Wound Dehiscence	0.14	0.03	1	1-1	0.11	20	16.5-23.8
Pneumonia	2.74	1.51	6	2-9	1.14	18	15-23.5
Reintubation	1.74	1.03	3	2-7.8	0.71	14	7-19.25
Urinary Tract Infection	2.14	1.00	5	0.5-6	1.09	13.5	9.25-21.5
Sepsis/Septic Shock	2.09	0.66	6	5-9.5	1.43	17	14-26
VTE	3.11	1.20	4.5	2-7.8	1.91	17	14-22.5
Cerebrovascular Accident	1.06	0.60	2	1-6	0.46	14	10-18.5
Cardiac Arrest/MI	0.43	0.17	0	0-0	0.26	20	18-24
Reoperation	4.86	2.49	3	2-7.5	2.37	16	12-23.5
Unplanned Readmission	12.20		–	–	12.20	17	11-23
Death	4.26	1.17	10	6-15	3.09	20	15-26

IQR, Interquartile range; MI, Myocardial infarction; VTE, venous thromboembolism.

Reoperation occurred in 5% of patients (n=170). Reoperations were equally likely to occur during index hospitalization as after discharge (**Figure 1B**). The most frequently coded reoperation within 30 days was hematoma evacuation (n=33, 1% of all patients), which occurred a median of 2 days post-operatively (IQR 0-6), followed by the placement of external ventricular drain (EVD) or cerebrospinal fluid (CSF) shunt (n=22, 0.6%), which occurred after a median of 3 days (range 0-6) and 13 days (IQR 6-16) respectively. Among hematoma evacuations, 28% occurred on the same day as the index procedure while another 21% occurred on the first post-operative day.

Unplanned readmission was observed in 12% of cases (n=427). The most frequent indications surgical site infections (SSI) (n=39, 1%), non-infectious cardiac or pulmonary diseases (n=28, 0.8%), oncological management of metastasis (n=28, 0.8%), and pneumonia (n=27, 0.8%).

Death within 30 days of a craniotomy occurred in 4% of patients and demonstrated wide temporal trends related to discharge. While the majority of 30-day deaths occurred after discharge (72%), over a quarter were within the index hospitalization. Amongst patients with tracked mortality in this cohort, 66% were male and the median age was 63 years (IQR: 55 – 71). These patients were sicker than those who did not die: 32% of patients who died within 30 days were frail compared to 16% of those who did not. Furthermore, death after discharge occurred in 7% of patients requiring a reoperation compared to 3% of patients who did not (OR 2.4, p = 0.006). Similarly, death after discharge occurred in 12% of readmitted patients compared with 2% of non-readmitted patients (OR 7.7, p < 0.001).

### Impact of Tumor Location

We hypothesized that tumor location might affect the risk for a post-operative complication and mortality. Patients with infratentorial metastasis demonstrated similar demographic and co-morbid characteristics as those with supratentorial metastases, including age, gender, BMI, prior smoking history, CHF, COPD, hypertension, and diabetes. Craniotomies for infratentorial lesions were associated with an average operative time 33 minutes longer than supratentorial approaches (p < 0.001).

**Figure 2:**
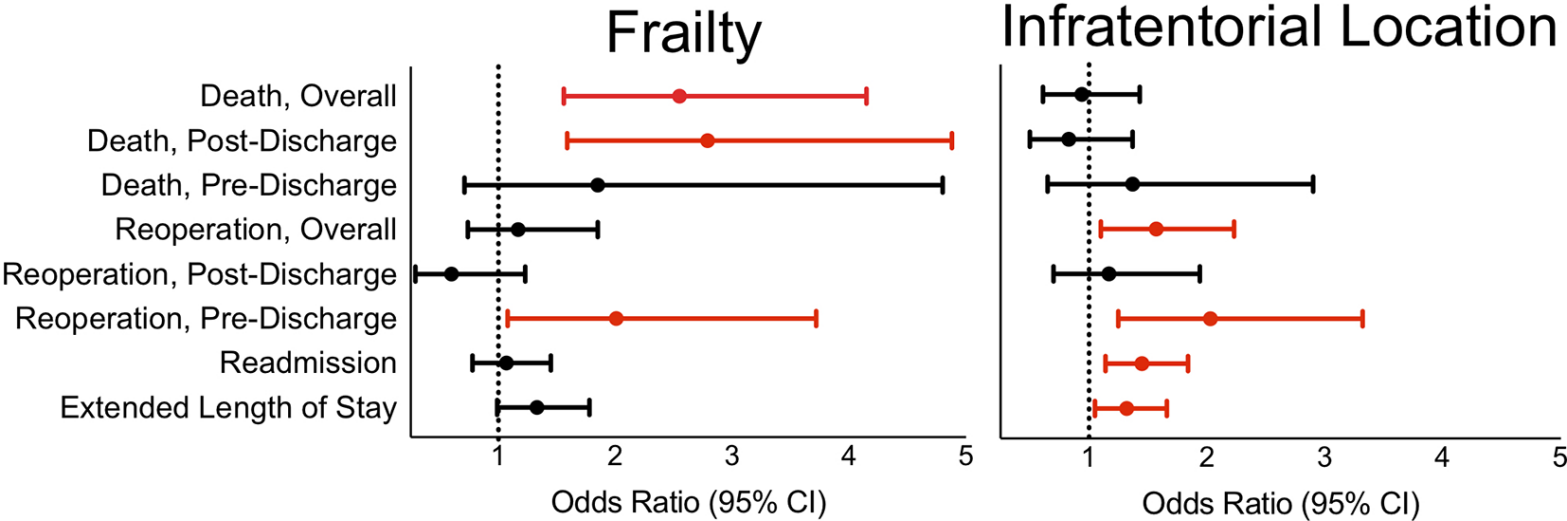
In reference to supratentorial approaches, infratentorial surgical approach is associated with length of stay over 7 days, 30-day overall and pre-discharge reoperation, and unplanned readmission. In reference to non-frailty, frailty is associated with 30-day pre-discharge reoperation as well as overall and post-discharge death.

Infratentorial location was found to be associated with increased odds for multiple 30-day medical complications, reoperation, and unplanned readmission (all p < 0.05) **(** **Table 3**). In particular, it was associated with increased odds of SSI (OR 2.9), pneumonia (OR 1.6), and reintubation (OR 1.9). It was also associated with length of stay above 7 days (OR 1.3) (**Figure 2**). Interestingly, infratentorial location was associated with decreased odds of VTE (OR 0.6).

**Table 3:** Adjusted association of infratentorial approach with 30-day postoperative complications.

	Total	Infratentorial	Supratentorial			
Medical Complications	%	%	%	OR	95% CI	p
Surgical Site Infection	1.49	2.90	1.05	2.92	1.62-5.28	<0.001
Wound Dehiscence	0.14	0.24	0.11	1.95	0.15-25.76	0.613
Pneumonia	2.74	3.99	2.36	1.63	1.01-2.63	0.047
Reintubation	1.74	3.02	1.34	1.90	1.08-3.33	0.026
Venous Thromboembolism	3.11	2.06	3.44	0.55	0.32-0.95	0.032
Urinary Tract Infection	2.14	1.93	2.13	0.83	0.46-1.49	0.529
Cerebrovascular Accident	1.06	1.09	1.05	0.81	0.36-1.79	0.597
Cardiac Arrest/MI	0.43	0.36	0.45	0.67	0.17-2.58	0.559
Transfusion	3.11	2.18	3.33	0.47	0.27-0.83	0.009
Sepsis/Septic Shock	2.09	2.42	1.98	1.19	0.68-2.09	0.534
Extended Length of Stay*	14.34	18.50	13.08	1.32	1.05-1.66	0.018
Reoperation	4.86	6.65	4.30	1.57	1.10-2.23	0.013
Pre-Discharge**	2.37	3.87	1.91	2.03	1.25-3.32	0.004
Post-Discharge	2.52	2.82	2.42	1.17	0.70-1.94	0.552
Hematoma Evacuation	0.94	0.97	0.93	1.24	0.52-2.96	0.633
EVD/CSF Shunt Placement	0.63	2.06	0.37	7.11	2.88-17.56	<0.001
Tumor Resection	0.57	0.73	0.52	1.86	0.65-5.30	0.247
Abscess Drainage	0.46	0.36	0.49	0.93	0.24-3.64	0.918
Unplanned Readmissions**	12.32	15.46	11.35	1.45	1.14-1.84	0.002
Surgical Site Infection	1.10	1.84	0.87	2.54	1.25-5.15	0.010
Cardiac/Pulm Disease	0.81	1.35	0.64	2.30	0.99-5.33	0.053
Brain Metastases	0.81	0.86	0.79	1.31	0.53-3.23	0.560
Pneumonia	0.78	0.61	0.83	0.90	0.32-2.54	0.837
Seizure	0.75	0.37	0.87	0.36	0.10-1.28	0.115
Primary Cancer	0.69	0.61	0.72	0.62	0.20-1.91	0.408
GI	0.72	0.98	0.64	1.72	0.70-4.21	0.237
VTE	0.66	0.86	0.61	1.38	0.51-3.74	0.522
CVA	0.58	0.49	0.61	0.82	0.24-2.76	0.745
Electrolyte/Metabolic	0.49	0.98	0.34	4.46	1.52-13.07	0.007
Sepsis/Septic Shock	0.38	0.12	0.45	0.27	0.03-2.23	0.225
Hydrocephalus	0.35	0.86	0.19	5.11	1.32-19.68	0.018
Death	4.26	4.11	4.30	0.94	0.61-1.43	0.765
Post-Discharge**	3.12	2.70	3.25	0.83	0.50-1.37	0.462
Pre-Discharge	1.17	1.45	1.08	1.37	0.65-2.90	0.405

*excluding include 4 missing data points.

**excluding 41 cases that died on index hospitalization.

CI, Confidence interval; CSF, Cerebrospinal fluid; CVA, Cerebrovascular Accident; EVD, External ventricular drain; GI, Gastrointestinal; MI, Myocardial Infarction; OR, Odds ratio; Pulm, Pulmonary; VTE, venous thromboembolism.

Focusing on those cases with reoperation, infratentorial location was associated specifically with reoperations during the index hospitalization but not after discharge **(** **Table 3** **)**. Amongst infratentorial lesions, 27% of reoperations were for CSF shunting (not including lumbar drains), 15% entailed evacuation of a hematoma, and 11% were for placement of EVD. Placement of EVD or CSF shunt after the initial craniotomy for tumor was significantly more likely for infratentorial metastases than supratentorial metastases (OR 7.1).

Concordantly, infratentorial location was associated with significant risk of unplanned readmission for hydrocephalus (OR 5.1) **(** **Table 3** **)**. The presence of hydrocephalus correlated with reoperation for CSF shunting or EVD placement for infratentorial lesions: 75% of hydrocephalus cases received either CSF shunt or EVD compared to 0.4% of cases without hydrocephalus (χ^2^, p < 0.001). Readmissions for electrolyte/metabolic derangements (OR 4.5) and surgical site infections (OR 2.5) were also more common following infratentorial approaches. There was no statistically significant association between tumor location and death (p = 0.77).

### Impact of Frailty

We further assessed the association of frailty, as a composite reflection of chronic disease burden, and postoperative outcomes. Frail patients had significantly higher likelihood of developing pneumonia (OR 2.8) **(** **Table 4** **)**. Frailty was also associated with increased risk of reoperation on the index hospitalization compared to non-frail patients (OR 2.0), but not reoperation after discharge (**Figure 2**). Frail patients were especially at risk for surgical evacuation of hematoma (OR 3.6). Frailty was not associated with unplanned readmission overall or any specific reasons for readmission. Pre-frailty and frailty had elevated odds ratios of 1.8 and 2.6 for death compared to non-frail patients, respectively. On temporal analysis, frailty was associated with death after discharge, but not before discharge.

**Table 4:** Adjusted association of pre-frailty and frailty with 30-day postoperative complications compared with non-frail patients.

	Non-Frail	Pre-Frail				Frail			
Medical Complications	%	%	OR	95% CI	p	%	OR	95% CI	p
Surgical Site Infection	1.91	0.88	0.43	0.21-0.90	0.026	1.54	0.80	0.34-1.86	0.600
Dehiscence	0.18	0.08	0.22	0.02-2.95	0.250	0.17	0.35	0.02-5.45	0.453
Pneumonia	1.91	2.17	1.02	0.59-1.79	0.933	6.35	2.78	1.59-4.87	<0.001
Reintubation	1.26	1.69	1.05	0.55-2.02	0.875	3.26	1.64	0.80-3.36	0.175
VTE	2.99	2.73	0.78	0.48-1.26	0.315	4.29	1.16	0.67-2.03	0.595
Urinary Tract Infection	1.38	2.49	1.52	0.84-2.73	0.167	3.26	1.56	0.78-3.14	0.212
Cerebrovascular Accident	0.66	1.53	1.84	0.82-4.14	0.141	1.20	1.13	0.39-3.26	0.827
Cardiac Arrest/MI	0.30	0.32	0.57	0.14-2.31	0.431	1.03	1.40	0.38-5.17	0.617
Transfusion	2.39	3.13	1.18	0.70-1.98	0.537	4.81	1.27	0.69-2.34	0.435
Sepsis/Septic Shock	1.44	2.17	1.18	0.65-2.14	0.587	3.77	1.64	0.84-3.20	0.145
Extended Length of Stay*	12.03	14.88	1.14	0.90-1.45	0.283	19.93	1.33	0.99-1.78	0.056
**Reoperation**	4.67	4.58	0.91	0.62-1.33	0.631	6.00	1.17	0.74-1.85	0.510
Pre-Discharge	1.91	2.17	1.13	0.64-1.97	0.679	4.12	2.01	1.08-3.72	0.027
Post-Discharge**	2.77	2.44	0.79	0.48-1.30	0.352	1.93	0.60	0.29-1.23	0.159
Hematoma Evacuation	0.54	1.20	2.63	1.04-6.60	0.040	1.54	3.58	1.24-10.32	0.018
EVD/CSF Shunt Placement	0.60	0.40	0.63	0.22-1.80	0.384	1.20	1.57	0.53-4.64	0.410
Tumor Resection	0.90	0.32	0.34	0.10-1.12	0.075	0.17	0.17	0.02-1.46	0.106
Abscess Drainage	0.66	0.24	0.30	0.07-1.24	0.097	0.34	0.58	0.10-3.24	0.537
**Unplanned Readmission**	11.51	12.46	0.97	0.76-1.24	0.823	14.36	1.07	0.78-1.45	0.686
Surgical Site Infection	1.39	0.73	0.45	0.19-1.06	0.067	1.05	0.75	0.27-2.09	0.581
Cardiac/Pulm Disease	0.54	0.73	0.94	0.34-2.62	0.913	1.75	2.37	0.84-6.72	0.104
Brain Metastases	0.78	0.98	1.29	0.56-3.00	0.553	0.53	0.73	0.19-2.83	0.647
Pneumonia	0.54	0.90	1.82	0.69-4.80	0.225	1.23	2.41	0.78-7.44	0.127
Seizure	0.66	0.73	0.81	0.31-2.13	0.665	1.05	1.24	0.39-3.96	0.713
Primary Cancer	0.66	0.98	1.37	0.53-3.53	0.513	0.18	0.17	0.02-1.53	0.113
GI	0.66	0.81	1.04	0.41-2.63	0.932	0.70	0.71	0.20-2.52	0.591
VTE	0.60	0.73	0.90	0.34-2.41	0.832	0.70	0.84	0.24-3.01	0.792
CVA	0.30	0.98	2.36	0.76-7.32	0.138	0.53	1.15	0.24-5.54	0.865
Electrolyte/Metabolic	0.54	0.41	0.70	0.21-2.29	0.554	0.53	0.90	0.21-3.82	0.892
Sepsis/Septic Shock	0.18	0.49	2.26	0.5-10.24	0.289	0.70	2.91	0.52-16.19	0.222
Hydrocephalus	0.30	0.41	0.93	0.23-3.69	0.916	0.35	0.40	0.04-3.67	0.419
**Death**	2.45	4.82	1.76	1.13-2.74	0.012	8.23	2.55	1.56-4.15	<0.001
Post-Discharge	1.75	3.50	1.72	1.04-2.87	0.036	6.30	2.79	1.59-4.88	<0.001
Pre-Discharge	0.72	1.37	1.75	0.74-4.09	0.200	2.06	1.85	0.71-4.80	0.208

*4 cases with missing length of stay not included.

**excluding 41 cases that died on index hospitalization.

CI, Confidence interval; CSF, Cerebrospinal fluid; CVA, Cerebrovascular Accident; EVD, External ventricular drain; GI, Gastrointestinal; MI, Myocardial Infarction; OR, Odds ratio; Pulm, Pulmonary; VTE, venous thromboembolism.

### Predictive Model for Pre-Mature Mortality

A multivariate logistic regression analysis with stepwise variable elimination was performed to construct a predictive model for death within 30 days after surgery for brain metastasis **(** **Table 5** **)**. Notably, frailty and pre-frailty were significant predictors in this model. Thrombocytopenia below 100,000 cells/dL had the highest strength of association of any variable. Additional predictors within the model included ASA score of 4/5, male sex, age over 75 years, other preoperative laboratory abnormalities, non-elective case designation, and preoperative steroid use. The model demonstrated a fair ability to correctly classify patients who die prematurely (AUROC 0.75).

**Table 5:** Multivariate logistic regression predictive model for 30-day death sorted by predictor effect size.

Predictor	OR	95% CI	p-value
Thrombocytopenia (under 100,000 cells/dL)	3.41	1.32 - 7.69	0.006
ASA score of 4-5	3.05	1.38 - 8.09	0.012
Frailty	2.48	1.59 - 3.89	<0.001
Male Sex	2.18	1.53 - 3.14	<0.001
Age 75+	2.00	1.24 - 3.12	0.003
WBCs above 12,000 cells/dL	1.88	1.33 - 2.66	<0.001
*ASA score of 3*	*1.79*	*0.83 - 4.66*	*0.179*
Anemia (hematocrit 24-36%)	1.73	1.20 - 2.46	0.003
Urgent/Emergent Case	1.68	1.17 - 2.43	0.005
Pre-frailty	1.62	1.09 - 2.50	0.021
Pre-operative Steroid Use	1.58	1.08 - 2.29	0.017
*Hyponatremia*	*1.40*	*0.93 - 2.08*	*0.098*
AUROC			0.754
Hosmer-Lemeshow Statistic			0.211

ASA, American Society of Anesthesiologists; CI, Confidence interval; OR, Odds ratio; WBC, White blood cells.

Predictors that did not meet criteria for statistical significance but that remained in the model after stepwise variable elimination are italicized.

## Discussion

Determining which patients will benefit from surgical resection of brain metastases requires balancing potential improvements in overall survival and local control with the risks of surgery, hospitalization, and rehabilitation. We provide a global perspective for this common diagnosis and a predictive tool for 30-day mortality risk.

Post-operative complications during the index hospitalization demonstrated clear temporal trends and did not fit neatly into the classic “wind, water, wound, walk” surgical teaching paradigm (23). This cohort experienced early, but infrequent cardiac events usually the day of operation; cerebrovascular accidents at a median 2 days post-operatively; UTIs and pneumonia at a median of 5-6 days post-operatively; and surgical site infections 2 weeks after operation, suggesting that SSIs may be more commonly related to post-operative seeding from sources such as bedding and dressing changes. Awareness of the peak incidence for these complications may highlight tailored screening and follow-up when clinically indicated. Infratentorial approaches for tumor resection were associated with higher rates of pneumonia and reintubation, which are pulmonary complications potentially attributed to aspiration events and dysphagia related to cranial nerve dysfunction (24–26).

Reoperations occurred in a minority of patients, with the most common reason for reoperation being evacuation of intracranial hematoma. This usually occurred in the perioperative period. We found that craniotomies for infratentorial pathologies were associated with reoperation for CSF diversion, with similar rates of this potential complication during the index hospitalization or after discharge, suggesting the need for vigilance against hydrocephalus in both early and delayed settings. Frailty was associated with higher risk of reoperation before discharge but not post-discharge, as well as risk for hematoma evacuation.

More so than for other neurosurgical patients, for those with guarded prognoses, attempts to maximize quality of life and time spent with family or out of the hospital become tantamount. Surgery for infratentorial lesions had increased odds of readmission overall and for readmission associated with hydrocephalus, electrolyte/metabolic derangements, and pneumonia. The association with hydrocephalus is consistent with the elevated odds of intracranial pressure-reducing surgery observed for infratentorial metastases. Further, the proximity of the third ventricle to the hypothalamus may explain the relationship between hydrocephalus and electrolyte anomalies previously observed. The reasons for the lack of association between frailty and readmission may be multifactorial, including bias in the cohort studied or the goals of care for brain metastases patients, for whom palliation may be considered over hospitalization when non-operative complications arise.

The incidence of 30-day death for brain metastases was similar to that reported in other series, and slightly higher than rates observed for other intracranial tumors (27–30). This rate is improved compared to in-hospital postoperative mortality of 3% for brain metastases observed from 1988-2000, which may reflect improved operative management, perioperative care, and patient selection (31). Death occurred more frequently after discharge, though only 1% of all patients died prior to discharge. Given the importance of assessing postoperative mortality risk, we developed a predictive model for 30-day mortality that revealed high ASA score, frailty, male sex, thrombocytopenia amongst other preoperative laboratory abnormalities, case urgency, and preoperative steroid usage to predict mortality. Certain factors included in this model, like frailty and ASA score, are proxies for well-being as well as chronic disease burden and increased nutritional and medical needs that are best met in the inpatient setting, leading to a deterioration after discharge. This tool may augment surgical experience for borderline cases. Importantly, factors that are included may not have a mechanistic link to the outcome but may be proxy metrics for correlates that were not assessed. Factors that are not included in a model may still be important to consider in the management of the patient, but do not provide an added benefit in the predictive capacity of an empiric model.

Providers managing patients after surgery should be cognizant of the expected time course for common perioperative complications and adjust surveillance and follow-up. While tumor location may not be a modifiable factor, monitoring after surgery is. Implementing protocols to avoid pulmonary complications and close monitoring for signs of increased intracranial pressure may mitigate the risks associated with the infratentorial location for example (32). Optimization of elements that contribute to frailty, including blood pressure and blood glucose, could further decrease the additional per-operative risks incurred by patients. Given the association between frailty and post-discharge death in particular, frail patients would benefit from an assessment of their understanding regarding postoperative and chronic medical needs, medications, and appointments. Involving patients’ family members and caregivers in their medical planning may assist in these efforts.

This study has pertinent limitations in methodology. We capture 3,500 cases, which is likely a small proportion of all brain metastasis resections within this time frame (2). The NSQIP database only encapsulates complications up to 30 days after operation, which is generally below the expected survival of brain metastasis patients and thus limits long-term predictions. Supratentorial tumors have a wide range of operative risk based on eloquence, language dominance and lobe, which is not captured in this study, and are important for further study. For example, a complication for a metastasis near the motor cortex may cause hemiplegia and significantly decrease quality of life, which would not be captured in these data. Hospitals are not required to report all surgeries that occur at their institution, thus selection bias can be introduced depending on those cases the hospital chooses to report. There may also be confounding by hospital type, size, and specialization as hospital-level data are not available. The NSQIP database does not contain oncologic parameters relevant to the care of brain metastasis patients, including size and number of metastases, symptomatology, neo-adjuvant chemo- or radiotherapy, and histology, and does not track patients’ quality of life and overall survival. There are no data included on post-operative thromboprophylaxis.

## Conclusion

Selecting candidates for brain metastasis resection deliberately balances anticipated benefits to quality of life and survival with post-operative risks and recovery time. Tumor location and patient frailty expose patients to differing post-operative risks and should be considered in personalized surgical decision-making. We highlight our predictive tool for 30-day mortality in these patients that incorporates frailty as well as other demographic and clinical factors.

## Data Availability Statement

The datasets presented in this study can be found in online repositories. The names of the repository/repositories and accession number(s) can be found below: https://www.facs.org/Quality-Programs/ACS-NSQIP.

## Ethics Statement

Ethical review and approval was not required for the study on human participants in accordance with the local legislation and institutional requirements. Written informed consent for participation was not required for this study in accordance with the national legislation and the institutional requirements.

## Author Contributions

The conceptualization and design of this work was performed by SG, HD, LF, EH, ID, and WB. Data extraction and preparation was performed by SG, AGL, HD, and LF. Data analysis was performed by SG, AG, HD, LF, and EH. Data interpretation was performed by all authors. Manuscript drafting was performed by SG, AG, HD, LF, EH, EL, AA, and WB. All authors contributed to the article and approved the submitted version.

## Supplementary Material

The Supplementary Material for this article can be found online at: https://www.frontiersin.org/articles/10.3389/fonc.2021.662943/full#supplementary-material

## Conflict of Interest 

The authors declare that the research was conducted in the absence of any commercial or financial relationships that could be construed as a potential conflict of interest.
