# Supplementary material for: Surgical and Peri-Operative Considerations for Brain Metastases
Source: Front Oncol. 2021 May 5;11:662943. doi: 10.3389/fonc.2021.662943 (PMC8131835; doi:10.3389/fonc.2021.662943)
Supplement: Supplementary file 1 [file Table_1.docx]

**Supplementary Material**

**Supplementary Material 1**: Adjusted association of infratentorial approach with 30-day postoperative complications

|  | **Total** | **Infratentorial** | **Supratentorial** |  |  |  |
| --- | --- | --- | --- | --- | --- | --- |
| **Medical Complications** | **%** | **%** | **%** | **OR** | **95% CI** | **p** |
| Surgical Site Infection | 1.49 | 2.90 | 1.05 | 2.92 | 1.62-5.28 | <0.001 |
| Wound Dehiscence | 0.14 | 0.24 | 0.11 | 1.95 | 0.15-25.76 | 0.613 |
| Pneumonia | 2.74 | 3.99 | 2.36 | 1.63 | 1.01-2.63 | 0.047 |
| Reintubation | 1.74 | 3.02 | 1.34 | 1.90 | 1.08-3.33 | 0.026 |
| Venous Thromboembolism | 3.11 | 2.06 | 3.44 | 0.55 | 0.32-0.95 | 0.032 |
| Urinary Tract Infection | 2.14 | 1.93 | 2.13 | 0.83 | 0.46-1.49 | 0.529 |
| Cerebrovascular Accident | 1.06 | 1.09 | 1.05 | 0.81 | 0.36-1.79 | 0.597 |
| Cardiac Arrest/MI | 0.43 | 0.36 | 0.45 | 0.67 | 0.17-2.58 | 0.559 |
| Transfusion | 3.11 | 2.18 | 3.33 | 0.47 | 0.27-0.83 | 0.009 |
| Sepsis/Septic Shock | 2.09 | 2.42 | 1.98 | 1.19 | 0.68-2.09 | 0.534 |
| **Extended Length of Stay*** | **14.34** | **18.50** | **13.08** | **1.32** | **1.05-1.66** | **0.018** |
| **Reoperation** | **4.86** | **6.65** | **4.30** | **1.57** | **1.10-2.23** | **0.013** |
| Pre-Discharge** | 2.37 | 3.87 | 1.91 | 2.03 | 1.25-3.32 | 0.004 |
| Post-Discharge | 2.52 | 2.82 | 2.42 | 1.17 | 0.70-1.94 | 0.552 |
| Hematoma Evacuation | 0.94 | 0.97 | 0.93 | 1.24 | 0.52-2.96 | 0.633 |
| EVD/CSF Shunt Placement | 0.63 | 2.06 | 0.37 | 7.11 | 2.88-17.56 | <0.001 |
| Tumor Resection | 0.57 | 0.73 | 0.52 | 1.86 | 0.65-5.30 | 0.247 |
| Abscess Drainage | 0.46 | 0.36 | 0.49 | 0.93 | 0.24-3.64 | 0.918 |
| Unplanned Readmissions** | **12.32** | **15.46** | **11.35** | **1.45** | **1.14-1.84** | **0.002** |
| Surgical Site Infection | 1.10 | 1.84 | 0.87 | 2.54 | 1.25-5.15 | 0.010 |
| Cardiac/Pulm Disease | 0.81 | 1.35 | 0.64 | 2.30 | 0.99-5.33 | 0.053 |
| Brain Metastases | 0.81 | 0.86 | 0.79 | 1.31 | 0.53-3.23 | 0.560 |
| Pneumonia | 0.78 | 0.61 | 0.83 | 0.90 | 0.32-2.54 | 0.837 |
| Seizure | 0.75 | 0.37 | 0.87 | 0.36 | 0.10-1.28 | 0.115 |
| Primary Cancer | 0.69 | 0.61 | 0.72 | 0.62 | 0.20-1.91 | 0.408 |
| GI | 0.72 | 0.98 | 0.64 | 1.72 | 0.70-4.21 | 0.237 |
| VTE | 0.66 | 0.86 | 0.61 | 1.38 | 0.51-3.74 | 0.522 |
| CVA | 0.58 | 0.49 | 0.61 | 0.82 | 0.24-2.76 | 0.745 |
| Electrolyte/Metabolic | 0.49 | 0.98 | 0.34 | 4.46 | 1.52-13.07 | 0.007 |
| Sepsis/Septic Shock | 0.38 | 0.12 | 0.45 | 0.27 | 0.03-2.23 | 0.225 |
| Hydrocephalus | 0.35 | 0.86 | 0.19 | 5.11 | 1.32-19.68 | 0.018 |
| **Death** | 4.26 | 4.11 | 4.30 | 0.94 | 0.61-1.43 | 0.765 |
| Post-Discharge** | 3.12 | 2.70 | 3.25 | 0.83 | 0.50-1.37 | 0.462 |
| Pre-Discharge | 1.17 | 1.45 | 1.08 | 1.37 | 0.65-2.90 | 0.405 |

*excluding include 4 missing data points

** excluding 41 cases that died on index hospitalization

CI: Confidence interval; CSF: Cerebrospinal fluid; CVA: Cerebrovascular Accident; EVD: External ventricular drain; GI: Gastrointestinal; MI: Myocardial Infarction; OR: Odds ratio; Pulm: Pulmonary; VTE: venous thromboembolism

**Supplementary Material 2.** Adjusted association of pre-frailty and frailty with 30-day postoperative complications compared with non-frail patients.

|  | **Non-Frail** | **Pre-Frail** | | | | **Frail** | | | |
| --- | --- | --- | --- | --- | --- | --- | --- | --- | --- |
| **Medical Complications** | **%** | **%** | **OR** | **95% CI** | **p** | **%** | **OR** | **95% CI** | **p** |
| Surgical Site Infection | 1.91 | 0.88 | 0.43 | 0.21-0.90 | 0.026 | 1.54 | 0.80 | 0.34-1.86 | 0.600 |
| Dehiscence | 0.18 | 0.08 | 0.22 | 0.02-2.95 | 0.250 | 0.17 | 0.35 | 0.02-5.45 | 0.453 |
| Pneumonia | 1.91 | 2.17 | 1.02 | 0.59-1.79 | 0.933 | 6.35 | 2.78 | 1.59-4.87 | <0.001 |
| Reintubation | 1.26 | 1.69 | 1.05 | 0.55-2.02 | 0.875 | 3.26 | 1.64 | 0.80-3.36 | 0.175 |
| VTE | 2.99 | 2.73 | 0.78 | 0.48-1.26 | 0.315 | 4.29 | 1.16 | 0.67-2.03 | 0.595 |
| Urinary Tract Infection | 1.38 | 2.49 | 1.52 | 0.84-2.73 | 0.167 | 3.26 | 1.56 | 0.78-3.14 | 0.212 |
| Cerebrovascular Accident | 0.66 | 1.53 | 1.84 | 0.82-4.14 | 0.141 | 1.20 | 1.13 | 0.39-3.26 | 0.827 |
| Cardiac Arrest/MI | 0.30 | 0.32 | 0.57 | 0.14-2.31 | 0.431 | 1.03 | 1.40 | 0.38-5.17 | 0.617 |
| Transfusion | 2.39 | 3.13 | 1.18 | 0.70-1.98 | 0.537 | 4.81 | 1.27 | 0.69-2.34 | 0.435 |
| Sepsis/Septic Shock | 1.44 | 2.17 | 1.18 | 0.65-2.14 | 0.587 | 3.77 | 1.64 | 0.84-3.20 | 0.145 |
| **Extended Length of Stay*** | 12.03 | 14.88 | 1.14 | 0.90-1.45 | 0.283 | 19.93 | 1.33 | 0.99-1.78 | 0.056 |
| **Reoperation** | 4.67 | 4.58 | 0.91 | 0.62-1.33 | 0.631 | 6.00 | 1.17 | 0.74-1.85 | 0.510 |
| Pre-Discharge | 1.91 | 2.17 | 1.13 | 0.64-1.97 | 0.679 | 4.12 | 2.01 | 1.08-3.72 | 0.027 |
| Post-Discharge** | 2.77 | 2.44 | 0.79 | 0.48-1.30 | 0.352 | 1.93 | 0.60 | 0.29-1.23 | 0.159 |
| Hematoma Evacuation | 0.54 | 1.20 | 2.63 | 1.04-6.60 | 0.040 | 1.54 | 3.58 | 1.24-10.32 | 0.018 |
| EVD/CSF Shunt Placement | 0.60 | 0.40 | 0.63 | 0.22-1.80 | 0.384 | 1.20 | 1.57 | 0.53-4.64 | 0.410 |
| Tumor Resection | 0.90 | 0.32 | 0.34 | 0.10-1.12 | 0.075 | 0.17 | 0.17 | 0.02-1.46 | 0.106 |
| Abscess Drainage | 0.66 | 0.24 | 0.30 | 0.07-1.24 | 0.097 | 0.34 | 0.58 | 0.10-3.24 | 0.537 |
| **Unplanned Readmission** | 11.51 | 12.46 | 0.97 | 0.76-1.24 | 0.823 | 14.36 | 1.07 | 0.78-1.45 | 0.686 |
| Surgical Site Infection | 1.39 | 0.73 | 0.45 | 0.19-1.06 | 0.067 | 1.05 | 0.75 | 0.27-2.09 | 0.581 |
| Cardiac/Pulm Disease | 0.54 | 0.73 | 0.94 | 0.34-2.62 | 0.913 | 1.75 | 2.37 | 0.84-6.72 | 0.104 |
| Brain Metastases | 0.78 | 0.98 | 1.29 | 0.56-3.00 | 0.553 | 0.53 | 0.73 | 0.19-2.83 | 0.647 |
| Pneumonia | 0.54 | 0.90 | 1.82 | 0.69-4.80 | 0.225 | 1.23 | 2.41 | 0.78-7.44 | 0.127 |
| Seizure | 0.66 | 0.73 | 0.81 | 0.31-2.13 | 0.665 | 1.05 | 1.24 | 0.39-3.96 | 0.713 |
| Primary Cancer | 0.66 | 0.98 | 1.37 | 0.53-3.53 | 0.513 | 0.18 | 0.17 | 0.02-1.53 | 0.113 |
| GI | 0.66 | 0.81 | 1.04 | 0.41-2.63 | 0.932 | 0.70 | 0.71 | 0.20-2.52 | 0.591 |
| VTE | 0.60 | 0.73 | 0.90 | 0.34-2.41 | 0.832 | 0.70 | 0.84 | 0.24-3.01 | 0.792 |
| CVA | 0.30 | 0.98 | 2.36 | 0.76-7.32 | 0.138 | 0.53 | 1.15 | 0.24-5.54 | 0.865 |
| Electrolyte/Metabolic | 0.54 | 0.41 | 0.70 | 0.21-2.29 | 0.554 | 0.53 | 0.90 | 0.21-3.82 | 0.892 |
| Sepsis/Septic Shock | 0.18 | 0.49 | 2.26 | 0.5-10.24 | 0.289 | 0.70 | 2.91 | 0.52-16.19 | 0.222 |
| Hydrocephalus | 0.30 | 0.41 | 0.93 | 0.23-3.69 | 0.916 | 0.35 | 0.40 | 0.04-3.67 | 0.419 |
| **Death** | 2.45 | 4.82 | 1.76 | 1.13-2.74 | 0.012 | 8.23 | 2.55 | 1.56-4.15 | <0.001 |
| Post-Discharge | 1.75 | 3.50 | 1.72 | 1.04-2.87 | 0.036 | 6.30 | 2.79 | 1.59-4.88 | <0.001 |
| Pre-Discharge | 0.72 | 1.37 | 1.75 | 0.74-4.09 | 0.200 | 2.06 | 1.85 | 0.71-4.80 | 0.208 |

* 4 cases with missing length of stay not included

** excluding 41 cases that died on index hospitalization

CI: Confidence interval; CSF: Cerebrospinal fluid; CVA: Cerebrovascular Accident; EVD: External ventricular drain; GI: Gastrointestinal; MI: Myocardial Infarction; OR: Odds ratio; Pulm: Pulmonary; VTE: venous thromboembolism
